# 纳米药物联合CAR-T疗法在实体肿瘤治疗中的研究进展

**DOI:** 10.3779/j.issn.1009-3419.2023.102.02

**Published:** 2023-01-20

**Authors:** Yi LIU, Ning LI, Wenyang JIANG, Qing GENG

**Affiliations:** 430060 武汉，武汉大学人民医院胸外科; Department of Thoracic Surgery, Renmin Hospital of Wuhan University, Wuhan 430060, China

**Keywords:** 纳米递送系统, 嵌合抗原受体T细胞疗法, 实体肿瘤, Nano delivery system, Chimeric antigen receptor T cell therapy, Solid tumors

## Abstract

嵌合抗原受体T细胞（chimeric antigen receptor T cell, CAR-T）疗法在血液系统肿瘤治疗中已取得巨大成功，但由于实体肿瘤特殊的肿瘤微环境和缺乏特异性的抗原靶点，CAR-T疗法在实体肿瘤中的治疗效果并不理想，联合其他疗法是目前的迫切需要。当前，纳米递送系统已经成为抗肿瘤药物研发最活跃的方向之一，本文在介绍CAR-T疗法与实体肿瘤治疗相关的背景基础上，重点关注纳米药物联合CAR-T疗法以增强肿瘤治疗的研究进展，从体内递送mRNA、调节肿瘤微环境、联合光热治疗等方面，较为系统地综述了近年来纳米递送系统联合CAR-T疗法的策略与典例，同时对该领域的未来方向进行展望。

## Abstract (en)

Chimeric antigen receptor T cell (CAR-T) therapy has shown remarkable success in treating hematological malignancies. However, CAR-T therapy for solid tumors is still limited due to the unique solid-tumor microenvironment and heterogeneous target antigen expression, which leads to an urgent need of combining other therapies. At present, nano delivery system has become one of the most promising directions for the development of anti-tumor drugs. Based on the background of CAR-T and tumor treatment, we focus on the research progress of nanomedicine combined with CAR-T therapy, and systematically review the strategies and examples in recent years in the aspects of in vivo delivery of mRNA, regulation of tumor microenvironment, combination with photothermal therapy. And we also look forward to the future direction of this filed.

## 1 嵌合抗原受体T细胞（chimeric antigen receptor T cell, CAR-T）疗法与实体肿瘤治疗

### 1.1 CAR-T疗法

CAR-T是一种过继细胞免疫疗法，其CAR结构包含细胞外抗原结合区、铰链区、跨膜区及细胞内信号传导区四部分，细胞外抗原结合区由识别肿瘤相关抗原的单链片段（single-chain variable fragment, scFv）组成，scFv可通过与肿瘤细胞上的特定靶抗原结合，刺激胞内信号传导分子CD3的免疫受体酪氨酸活化基序（immunoreceptor tyrosine-based activation motifs, ITAMs），从而激活T细胞活化增殖，杀伤肿瘤细胞^[1]^。迄今为止，CAR-T技术已更新至第四代（图1），第二代CAR-T已获批应用于临床，在血液系统肿瘤中疗效显著，如以CD19为靶点的CAR-T疗法可显著缓解急性淋巴细胞白血病的症状，有临床数据^[2]^显示其完全缓解（complete response, CR）率可达80%。

**图1:**
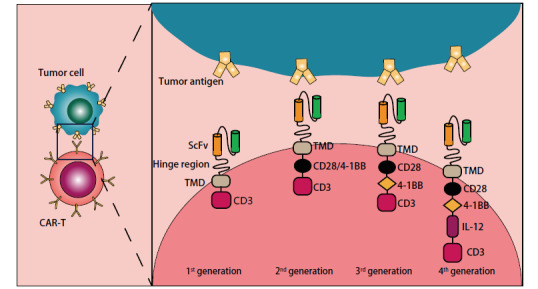
四代CAR-T的结构对比。第二代CAR-T在常规CAR上添加共刺激分子CD28或4-1BB以提升CAR-T增殖活化效能；第三代CAR-T同时添加两个共刺激分子；第四代CAR-T在第二、三代CAR基础上共表达IL-12等细胞因子，以增加CAR-T扩增及其他免疫杀伤细胞向肿瘤组织浸润。

### 1.2 CAR-T疗法在实体肿瘤中的挑战

尽管CAR-T疗法在血液系统肿瘤治疗中已经取得瞩目成就，但在实体肿瘤中的治疗效果仍不理想，限制其疗效的因素主要有以下几个方面。

#### 1.2.1 肿瘤微环境

肿瘤细胞命运受到肿瘤微环境调控，肿瘤细胞与肿瘤微环境间的交互作用维持并促进了微环境的药物抵抗和免疫抑制状态。肿瘤组织间质高压^[3]^、缺氧及酸性微环境^[4]^构成了肿瘤治疗的物理屏障，这种物理屏障限制了CAR-T细胞的浸润和扩增，导致CAR-T治疗效果不佳。

肿瘤细胞与免疫系统的交互作用促进了免疫抑制微环境。如肿瘤细胞表面上调的程序性细胞死亡配体1（programmed cell death ligand 1, PD-L1）与T细胞表面的程序性细胞死亡受体1（programmed cell death 1, PD-1）结合后，可使T细胞衰竭和功能障碍，导致肿瘤细胞的免疫逃逸。值得注意的是，单独接受PD-1/PD-L1抑制剂的患者中，60%会出现不良反应^[5]^，而使用CAR-T与PD-1抑制剂可显著提升CAR-T细胞的抗肿瘤效果并延长CAR-T细胞的工作寿命^[6]^。此外，随着肿瘤的进展，肿瘤微环境的一些细胞还发挥免疫负性调节作用，如肿瘤相关巨噬细胞（tumor associated macrophage, TAM）和调节性T细胞（regulatory T cells, Treg）^[7]^，实体肿瘤组织内的TAM多表现为M2表型，TAM可分泌白介素（interleulin, IL）-8、血管内皮生长因子（vascular endothelial growth factor, VEGF）等直接促进肿瘤浸润和转移的细胞因子，在与CAR-T共培养后，可显著限制CAR-T分泌γ干扰素（interferon γ, IFN-γ）的能力^[8]^。Treg则可通过分泌转化生长因子β（transforming growth factor β, TGF-β）等抑制免疫反应，从而限制CAR-T疗效。近年来，纳米药物靶向清除和重极化TAM已成为肿瘤免疫治疗的热点之一，尽管目前CAR-T联合靶向调控TAM的纳米药物的研究仍较少，但这有可能成为未来肿瘤治疗的重要策略。

#### 1.2.2 细胞因子风暴

CAR-T细胞回输到患者体内，在肿瘤细胞和免疫系统作用下激活扩增，机体会释放大量细胞因子，导致细胞因子释放综合征（cytokine release syndrome, CRS）和免疫效应细胞相关神经毒性综合征（immune effector cell-associated neurotoxicity syndrome, ICANS）等毒副作用^[9]^。值得注意的是，这些细胞因子并非完全由CAR-T释放，有证据^[10,11]^表明，CRS和ICANS更大程度上由巨噬细胞释放的IL-1和IL-6介导，CAR-T介导的肿瘤细胞凋亡所释放的因子也参与了细胞因子风暴的发生与发展^[12]^。在CAR-T治疗实体肿瘤的几项临床研究^[13⇓-15]^中，均观察到了不同程度的CRS，同时在脑胶质瘤中发现了ICANS^[15]^，因此，控制细胞因子释放也是未来CAR-T疗法改进的方向之一。尽管阻断IL-1、IL-6已被证明可改善细胞因子风暴，但是由于多种细胞因子之间存在复杂串扰，阻断特定细胞因子的效果并不理想，限制CAR-T在体内的循环时间是解决细胞因子释放的有效手段。虽然CAR-T数量在体内逐渐衰减，但细胞因子风暴的发生先于其衰减，通过纳米递送系统瞬时生成CAR-T，以减少CAR-T在体内循环时间，将为解决细胞因子风暴提供新思路。

#### 1.2.3 脱靶效应

CAR-T主要靶向作用于肿瘤相关抗原，而由于肿瘤相关抗原在人体其他组织上也存在表达，因此CAR-T可能攻击表达靶抗原的正常组织，造成脱靶效应，如碳酸酐酶IX在肾癌及正常胆管上皮上均存在表达，对肾细胞癌患者应用靶向碳酸酐酶IX的CAR-T治疗将导致患者出现肝功能损伤^[16]^。此外，随着肿瘤进展，肿瘤微环境与肿瘤细胞状态发生动态变化，这可能使肿瘤组织靶点下调并增加耐药性^[17]^，导致仅有一部分细胞群体表达CAR所靶向的抗原，从而限制CAR-T的治疗效果。提升CAR-T靶向性和减少CAR-T循环时间均可减轻脱靶效应，目前应对CAR-T脱靶效应的方法仍以寻找更具特异性的肿瘤相关抗原和设计多靶点CAR-T为主，而纳米药物在靶向性和制备瞬时CAR-T方面具有独特优势，为解决脱靶效应提供了新的方法。

## 2 纳米药物联合CAR-T用于实体肿瘤治疗

### 2.1 纳米脂质体递送mRNA

mRNA作为携带蛋白质信息的信使，进入细胞质后可翻译为目的蛋白。基于这一特性，可以在体外递送mRNA至细胞以表达目的蛋白，已有研究^[18,19]^尝试在体外通过向T细胞导入mRNA，以构建CAR-T细胞，在临床研究中这种CAR-T表现出良好的肿瘤杀伤效果，由于mRNA在T细胞内逐渐降解，其表达的CAR也仅在T细胞内短暂表达，因此这种非持续存在的CAR-T一定程度上避免了细胞因子风暴和脱靶效应。然而，这种体外构建CAR-T的方法需要通过电穿孔将mRNA导入T细胞，不仅操作困难，对T细胞也具有一定毒性，因此需要更安全高效的体内靶向递送mRNA的方法。由于mRNA不稳定，容易降解，并且在体内不能靶向地递送到目的细胞，所以需要对mRNA进行必要的修饰和选择合适的递送系统。纳米脂质体（lipid nanoparticle, LNP）具有安全性高、成本低等特点，是目前应用最广泛的mRNA递送系统，如在新型冠状病毒流行的背景下，LNP已用于递送病毒抗原mRNA疫苗，近期的临床数据^[20]^已验证了这种递送系统的安全性和有效性。

CAR-T细胞的常规制备方法需要从患者体内提取出T细胞，在体外进行基因编辑，但这种方法步骤复杂、成本高昂，LNP递送系统的发展为CAR-T体内制备提供了新方向。CD3分子是T细胞表面的特异性抗原，通过在LNP表面融合CD3抗体（aCD3-LNP），并在LNP中包载目的蛋白的mRNA，即可实现在体内生成CAR-T细胞的目的。Kheirolomoom等^[21]^发现，包载荧光蛋白mCherry的mRNA的aCD3-LNP可在体内特异性地转染T细胞，使之表达mCherry蛋白，并且转染后的T细胞还可浸润到小鼠肿瘤组织中，这验证了LNP在体内转染T细胞治疗肿瘤的可行性。在心肌纤维化中，aCD3-LNP递送系统同样可在体内制备靶向肌成纤维细胞的CAR-T细胞，这种CAR-T细胞可抑制肌成纤维细胞的活动，从而缓解和逆转心肌纤维化^[22]^。尽管上述研究验证了通过aCD3-LNP在体内合成CAR-T的可行性，但mRNA在细胞中会逐渐降解，CAR-T细胞并不能持续性存在。另外，在实体肿瘤免疫抑制的微环境中，常规方式注射的CAR-T细胞数量会逐渐衰减^[23]^，而重复注射CAR-T细胞存在价格昂贵等缺点，因此，未来的研究可聚焦以下两个方向：（1）提升CAR mRNA表达的持久性；（2）探究是否可通过重复给予aCD3-LNP以改善CAR-T数量衰减的问题。

mRNA的LNP递送系统还可应用于协同刺激CAR-T的活化增殖。CAR-T细胞的活化增殖依赖于抗原识别与免疫系统的活化信号，在实体肿瘤微环境中，由于结合肿瘤抗原后缺乏增殖信号，CAR-T细胞难以发挥肿瘤杀伤作用。封闭蛋白6（recombinant claudin 6, CLDN-6）是一种高特异性的肿瘤相关抗原，靶向CLDN-6的CAR-T细胞具有良好的抗肿瘤效果，Reinhard等^[24]^发现，在抗原提呈细胞（antigen-presenting cell, APC）中表达CLDN-6以刺激CAR-T启动免疫激活程序和扩增，构建包载CLDN-6 mRNA的纳米脂质体疫苗（CARVac）并转染树突状细胞（dentric cells, DCs）后，DCs可促进CAR-T细胞增殖及强激活，并提升CAR-T的肿瘤细胞溶解活性，相较于只应用CAR-T的荷瘤小鼠而言，联合应用CAR-T和CARVac具有更佳的抗肿瘤效果，并且不出现明显的CRS。目前，这种联合CARVac和CAR-T的BNT211疗法已进入临床试验阶段，有数据^[25]^表明在纳入的16例晚期实体瘤患者中，BNT211治疗6周后客观缓解率可达43%，并且伴随的CRS整体可控。以上结果可知，由于mRNA疫苗构建的CAR表达时间更短，可降低CAR-T脱靶风险和细胞因子风暴，现有的临床试验也取得了令人鼓舞的结果，相信这种疗法未来可一定程度上优化当前的实体肿瘤治疗格局。

### 2.2 调节肿瘤免疫微环境

实体肿瘤微环境处于免疫抑制状态，因此CAR-T疗法的效果通常并不理想，研究者最直接的策略是给予小分子药物或生物大分子来正向调控免疫微环境，从而达到促进肿瘤治疗的目的。然而直接给药的方式缺乏靶向性，通常引起全身反应，搭载特定药物或生物分子的纳米递送系统具有增加药物循环时间，提升药物的溶解度、生物利用度和主动和被动靶向性，从而提升肿瘤免疫治疗的效果的优点^[26]^。

PI3K信号通路的异常激活在肿瘤进展中起核心作用，以往的研究^[27]^发现，应用PI3K的选择性抑制剂PI-3065可破坏Treg细胞的作用，进而活化肿瘤免疫微环境。在肿瘤治疗中，通过纳米缓释系统搭载免疫调节剂通常具有更好的免疫调控效果，因此，Zhang等^[28]^构建了靶向肿瘤细胞的纳米脂质体，在该纳米脂质体中搭载了PI-3065和自然杀伤（natural killer, NK）T细胞的激活剂7DW8-5，体内实验中发现，该纳米脂质体可在肿瘤微环境中缓慢释放PI-3065和7DW8-5，小鼠肿瘤微环境中TAM细胞、Treg细胞等免疫抑制性细胞的数量显著减少，而NK T细胞和CD8^+ ^T细胞等免疫活化细胞数量则显著增加。这种重塑的免疫微环境维持2周左右，在该时间窗内CAR-T细胞可以顺利进入肿瘤组织内活化扩增，以杀伤肿瘤细胞并增强CAR-T的治疗效果。

免疫检查点阻断疗法也可重塑肿瘤免疫微环境，从而促进CAR-T治疗效果。然而免疫检查点阻断所需的单克隆抗体作为一种生物大分子，在体内缺乏靶向性，进入体内后难以在肿瘤组织内富集。纳米材料也可实现生物大分子的靶向递送，如Nie等^[29]^开发了一种磁性纳米簇，在该纳米簇上通过pH敏感的苯甲酸亚胺键连接PD-1抗体。构建过表达PD-1的细胞毒性T细胞并与这种纳米簇共培养，得到连接纳米簇的T细胞，由于该纳米簇具有磁性，在磁共振成像（magnetic resonance imaging, MRI）引导下，T细胞和纳米簇可靶向肿瘤组织，在肿瘤酸性环境中，苯甲酸亚胺键断裂，PD-1抗体释放。PD-1抗体在肿瘤的靶向释放重塑了免疫微环境，同时T细胞发挥抗肿瘤杀伤作用，实现了靶向的免疫检查点阻断治疗和过继细胞治疗的联合。实体肿瘤中Fas下调也是肿瘤细胞免疫逃逸的重要机制，基于LNP的良好递送能力，也有研究^[30]^通过将包载Fas质粒的LNP递送至肿瘤细胞，使肿瘤细胞Fas表达恢复，以增强过继T细胞的肿瘤杀伤作用。以上结果可知，纳米药物可作为肿瘤微环境的免疫活化剂，以增强CAR-T对肿瘤细胞的杀伤效果，遗憾的是，尽管目前已有纳米药物调控肿瘤微环境的临床试验正在开展，但联合CAR-T的临床研究仍存在较大空缺，肿瘤微环境具有高度的复杂性和动态变化性，未来还需在基础研究中进一步完善探究更具普适性的肿瘤微环境纳米干预药物，以促进靶向肿瘤微环境的纳米药物联合CAR-T疗法的临床转化。

### 2.3 基于纳米颗粒的光热疗法

（photothermal therapy, PTT）联合CAR-T 肿瘤的PTT是近来倍受关注的治疗方法，其将具有光热转换特性的材料注射到人体，利用靶向识别技术聚集到肿瘤组织，在体外光源的照射下，光热材料产热“烧死”肿瘤细胞^[31]^。值得注意的是，多项研究^[32,33]^表明，PTT不仅可以杀死肿瘤细胞以释放出更多的抗原物质和细胞因子，还可破坏肿瘤组织内细胞外基质，促进免疫活性细胞向肿瘤浸润，从而重塑肿瘤微环境并提升免疫治疗的效果。

为了探究PTT后是否可以促进CAR-T的治疗效果，Chen等^[34]^在小鼠黑色素瘤模型中进行瘤内注射光热物质治疗后，静脉注射靶向黑色素瘤的CAR-T细胞，发现PTT可以促进CAR-T向肿瘤浸润、增殖及活化，并且表现出更佳的抗肿瘤效果。而在大部分肿瘤疾病中，瘤内注射具有创伤性，并且可能引起肿瘤扩散，因此Zhu等^[35]^开发了一种纳米酶，这种纳米酶以纳米硫化铜作为框架，添加透明质酸（hyaluronic acid, HA）以靶向肿瘤细胞CD33抗原，在近红外光照射下，这种纳米酶的温度显著上升，使硫化铜框架表现出过氧化物酶样催化活性。在体内，纳米酶的光热-酶效应在肿瘤组织局部产生大量活性氧（reactive oxygen species, ROS），ROS破坏细胞外基质与增加血液灌注，从而重塑肿瘤微环境。进一步研究^[35]^发现，在非小细胞肺癌小鼠模型中，这种纳米酶的应用促进了CAR-T的治疗效果，免疫荧光结果也提示了CAR-T浸润到肿瘤组织的效果得到显著增强。

由于CAR-T细胞具有靶向肿瘤的能力，因此也有研究团队探究通过CAR-T细胞膜包裹光热物质以实现靶向PTT的可行性，如Ma等^[36]^制备的一种用CAR-T细胞膜包被的多孔纳米颗粒，在该系统中，细胞膜来源于已经被证实具有靶向清除肝癌细胞能力的CAR-T细胞，并在CAR-T细胞膜包被的多孔纳米材料上搭载光热剂IR780，该系统可以在CAR-T细胞膜作用下靶向肝癌细胞，通过光热效应清除肿瘤细胞，在小鼠皮下肝癌模型中可使肿瘤体积缩减50%。此外，具有光热性质的纳米颗粒也可用于提升CAR-T的靶向性，Miller等^[37]^在CAR结构基因上游添加了热休克蛋白转录起始序列，使工程化T细胞只在高温环境下表达CAR，近红外光照射后，在体内聚集在肿瘤组织的纳米光热粒子产热，可促进肿瘤组织附近的工程化T细胞表达转换成为CAR-T，从而提升CAR-T的靶向性。纳米光遗传学也正逐步应用到提升CAR-T靶向性中，Nguyen等^[38]^将CAR结构的功能结构域分离，并在分离的功能结构域上添加光响应模块，在转换纳米材料发射的特定波长的作用下，即可使CAR分离的功能结构域再融合，从而实现CAR-T在肿瘤组织内的靶向生成。目前，PTT联合CAR-T疗法已在临床前研究中展现了良好的抗肿瘤效果，随着近年来低温PTT策略的应用和光遗传学的进展，可以预见未来PTT联合CAR-T疗法会在肿瘤临床治疗中发挥重要作用。

**图2:**
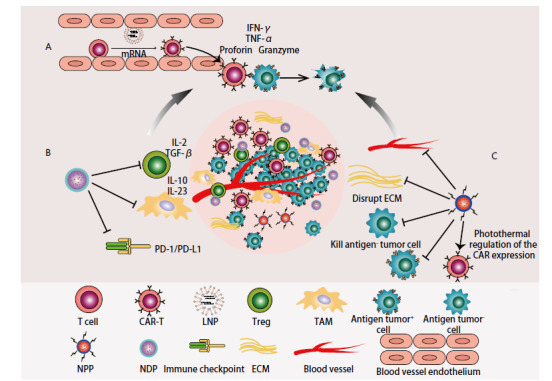
纳米药物联合CAR-T疗法的主要策略。A：通过纳米脂质体递送mRNA以在体内生成CAR-T；B：纳米递送系统靶向调控肿瘤免疫抑制微环境；C：光热疗法破坏肿瘤微环境物理屏障、杀伤抗原阴性肿瘤细胞，以及通过光热开关调控CAR表达。

## 3 总结与展望

综上，本文总结了纳米药物联合CAR-T疗法在实体肿瘤治疗中的最新策略与进展。目前，CAR-T疗法在实体肿瘤中的临床应用还处于探索阶段，以往的研究着重于关注提升CAR-T自身的效能，如优化CAR结构和寻找特异性的抗原靶点，然而单一应用CAR-T的疗效仍受到免疫抑制微环境和肿瘤细胞异质性等因素的限制，联用其他药物是目前采取的常规策略，但药物的靶向性和生物利用率是亟待解决的问题，纳米材料在靶向改善肿瘤免疫微环境和递送化疗、PTT药物方面均具有巨大优势，可提升CAR-T治疗的有效性。

然而，尽管CAR-T联合纳米药物较单一CAR-T疗法抗肿瘤效果更好，不良反应更小，但大部分研究还停留在基础研究阶段，临床研究仍旧缺乏，目前还需进一步探究这种联合疗法在动物和人体间的作用差异。当前CAR-T联合纳米药物的策略中使用的纳米材料还存在设计复杂、难以实现工业化生产的问题，进而限制了进一步的临床转化。因此，高效可行的治疗方案仍需进一步探索。此外，优化纳米材料自身理化性质，以提升药物负载效率、细胞及组织靶向性和生物相容性也是未来的重要方向。相信随着纳米医学的高速发展，纳米药物联合CAR-T的治疗策略有望为实体肿瘤开创一个极具应用前景的新型治疗局面。

## References

[1] Yu S, Li A, Liu Q, et al. Chimeric antigen receptor T cells: a novel therapy for solid tumors. J Hematol Oncol, 2017, 10(1): 78. doi: 10.1186/s13045-017-0444-9 28356156 PMC5372296

[2] Anagnostou T, Riaz IB, Hashmi SK, et al. Anti-CD19 chimeric antigen receptor T-cell therapy in acute lymphocytic leukaemia: a systematic review and meta-analysis. Lancet Haematol, 2020, 7(11): e816 -e826. doi: 10.1016/S2352-3026(20)30277-5 10.1016/S2352-3026(20)30277-5 33091355

[3] Minchinton AI, Tannock IF. Drug penetration in solid tumours. Nat Rev Cancer, 2006, 6(8): 583-592. doi: 10.1038/nrc1893 16862189

[4] Jin MZ, Jin WL. The updated landscape of tumor microenvironment and drug repurposing. Signal Transduct Target Ther, 2020, 5(1): 166. doi: 10.1038/s41392-020-00280-x 32843638 PMC7447642

[5] Zhou X, Yao Z, Bai H, et al. Treatment-related adverse events of PD-1 and PD-L1 inhibitor-based combination therapies in clinical trials: a systematic review and meta-analysis. Lancet Oncol, 2021, 22(9): 1265-1274. doi: 10.1016/S1470- 2045(21)00333-8 10.1016/S1470-2045(21)00333-8 34391508

[6] Adusumilli PS, Zauderer MG, Rivière I, et al. A phase I trial of regional mesothelin-targeted CAR T-cell therapy in patients with malignant pleural disease, in combination with the anti-PD-1 agent pembrolizumab. Cancer Discov, 2021, 11(11): 2748-2763. doi: 10.1158/2159-8290.CD-21-0407 34266984 10.1158/2159-8290.CD-21-0407 PMC8563385

[7] Galon J, Bruni D. Approaches to treat immune hot, altered and cold tumours with combination immunotherapies. Nat Rev Drug Discov, 2019, 18(3): 197-218. doi: 10.1038/s41573-018-0007-y 30610226

[8] Rodriguez-Garcia A, Lynn RC, Poussin M, et al. CAR-T cell-mediated depletion of immunosuppressive tumor-associated macrophages promotes endogenous antitumor immunity and augments adoptive immunotherapy. Nat Commun, 2021, 12(1): 877. doi: 10.1038/s41467-021-20893-2 33563975 PMC7873057

[9] Rafiq S, Hackett CS, Brentjens RJ. Engineering strategies to overcome the current roadblocks in CAR T cell therapy. Nat Rev Clin Oncol, 2020, 17(3): 147-167. doi: 10.1038/s41571-019-0297-y 31848460 PMC7223338

[10] Norelli M, Camisa B, Barbiera G, et al. Monocyte-derived IL-1 and IL-6 are differentially required for cytokine-release syndrome and neurotoxicity due to CAR T cells. Nat Med, 2018, 24(6): 739-748. doi: 10.1038/s41591-018-0036-4 29808007

[11] Giavridis T, van der Stegen SJC, Eyquem J, et al. CAR T cell-induced cytokine release syndrome is mediated by macrophages and abated by IL-1 blockade. Nat Med, 2018, 24(6): 731-738. doi: 10.1038/s41591-018-0041-7 29808005 PMC6410714

[12] Liu Y, Fang Y, Chen X, et al. Gasdermin E-mediated target cell pyroptosis by CAR T cells triggers cytokine release syndrome. Sci Immunol, 2020, 5(43): eaax7969. doi: 10.1126/sciimmunol.aax7969 31953257

[13] Narayan V, Barber-Rotenberg JS, Jung IY, et al. PSMA-targeting TGFβ-insensitive armored CAR T cells in metastatic castration-resistant prostate cancer: a phase 1 trial. Nat Med, 2022, 28(4): 724-734. doi: 10.1038/s41591-022-01726-1 35314843 PMC10308799

[14] Qi C, Gong J, Li J, et al. Claudin18.2-specific CAR T cells in gastrointestinal cancers: phase 1 trial interim results. Nat Med, 2022, 28(6): 1189-1198. doi: 10.1038/s41591-022-01800-8 35534566 PMC9205778

[15] Majzner RG, Ramakrishna S, Yeom KW, et al. GD2-CAR T cell therapy for H3K27M-mutated diffuse midline gliomas. Nature, 2022, 603(7903): 934-941. doi: 10.1038/s41586-022-04489-4 35130560 PMC8967714

[16] Lamers CH, Sleijfer S, Van Steenbergen S, et al. Treatment of metastatic renal cell carcinoma with CAIX CAR-engineered T cells: clinical evaluation and management of on-target toxicity. Mol Ther, 2013, 21(4): 904-912. doi: 10.1038/mt.2013.17 23423337 PMC5189272

[17] Raghavan S, Winter PS, Navia AW, et al. Microenvironment drives cell state, plasticity, and drug response in pancreatic cancer. Cell, 2021, 184(25): 6119-6137.e26. doi: 10.1016/j.cell.2021.11.017 34890551 PMC8822455

[18] Beatty GL, Haas AR, Maus MV, et al. Mesothelin-specific chimeric antigen receptor mRNA-engineered T cells induce anti-tumor activity in solid malignancies. Cancer Immunol Res, 2014, 2(2): 112-120. doi: 10.1158/2326-6066 24579088 PMC3932715

[19] Tchou J, Zhao Y, Levine BL, et al. Safety and efficacy of intratumoral injections of chimeric antigen receptor (CAR) T cells in metastatic breast cancer. Cancer Immunol Res, 2017, 5(12): 1152-1161. doi: 10.1158/2326-6066.CIR-17-0189 29109077 PMC5712264

[20] Li J, Hui A, Zhang X, et al. Safety and immunogenicity of the SARS-CoV-2 BNT162b1 mRNA vaccine in younger and older Chinese adults: a randomized, placebo-controlled, double-blind phase 1 study. Nat Med, 2021, 27(6): 1062-1070. doi: 10.1038/s41591-021-01330-9 33888900

[21] Kheirolomoom A, Kare AJ, Ingham ES, et al. In situ T-cell transfection by anti-CD3-conjugated lipid nanoparticles leads to T-cell activation, migration, and phenotypic shift. Biomaterials, 2022, 281: 121339. doi: 10.1016/j.biomaterials.2021.121339 35078042 PMC8892572

[22] Rurik JG, Tombácz I, Yadegari A, et al. CAR T cells produced in vivo to treat cardiac injury. Science, 2022, 375(6576): 91-96. doi: 10.1126/science.abm0594 34990237 PMC9983611

[23] O’rourke DM, Nasrallah MP, Desai A, et al. A single dose of peripherally infused EGFRvIII-directed CAR T cells mediates antigen loss and induces adaptive resistance in patients with recurrent glioblastoma. Sci Transl Med, 2017, 9(399): eaaa0984. doi: 10.1126/scitranslmed.aaa0984 28724573 PMC5762203

[24] Reinhard K, Rengstl B, Oehm P, et al. An RNA vaccine drives expansion and efficacy of claudin-CAR-T cells against solid tumors. Science, 2020, 367(6476): 446-453. doi: 10.1126/science.aay5967 31896660

[25] Haanen JBAG, Mackensen A, Koenecke C, et al. Abstract CT002: BNT211: A Phase I trial to evaluate safety and efficacy of CLDN6 CAR-T cells and CARVac-mediated in vivo expansion in patients with CLDN6-positive advanced solid tumors. Cancer Res, 2022, 82(12_Supplement): CT002. doi: 10.1158/1538-7445.AM2022-CT002

[26] Mu W, Chu Q, Liu Y, et al. A review on nano-based drug delivery system for cancer chemoimmunotherapy. Nanomicro Lett, 2020, 12(1): 142. doi: 10.1007/s40820-020-00482-6 34138136 PMC7770879

[27] Ali K, Soond DR, Pineiro R, et al. Inactivation of PI(3)K p110δ breaks regulatory T-cell-mediated immune tolerance to cancer. Nature, 2014, 510(7505): 407-411. doi: 10.1038/nature13444 24919154 PMC4501086

[28] Zhang F, Stephan SB, Ene CI, et al. Nanoparticles that reshape the tumor milieu create a therapeutic window for effective T-cell therapy in solid malignancies. Cancer Res, 2018, 78(13): 3718-3730. doi: 10.1158/0008-5472.CAN-18-0306 29760047 PMC6030470

[29] Nie W, Wei W, Zuo L, et al. Magnetic nanoclusters armed with responsive PD-1 antibody synergistically improved adoptive T-cell therapy for solid tumors. ACS Nano, 2019, 13(2): 1469-1478. doi: 10.1021/acsnano.8b07141 30763076

[30] Al Subeh ZY, Poschel DB, Redd PS, et al. Lipid nanoparticle delivery of fas plasmid restores fas expression to suppress melanoma growth in vivo. ACS Nano, 2022, 16(8): 12695-12710. doi: 10.1021/acsnano.2c04420 35939651 PMC9721370

[31] Chen Q, Liang C, Wang C, et al. An imagable and photothermal “Abraxane-like” nanodrug for combination cancer therapy to treat subcutaneous and metastatic breast tumors. Adv Mater, 2015, 27(5): 903-910. doi: 10.1002/adma.201404308 25504416

[32] Ren H, Yong J, Yang Q, et al. Self-assembled FeS-based cascade bioreactor with enhanced tumor penetration and synergistic treatments to trigger robust cancer immunotherapy. Acta Pharm Sin B, 2021, 11(10): 3244-3261. doi: 10.1016/j.apsb.2021.05.005 34729313 PMC8546854

[33] Zhang Y, Wang T, Tian Y, et al. Gold nanorods-mediated efficient synergistic immunotherapy for detection and inhibition of postoperative tumor recurrence. Acta Pharm Sin B, 2021, 11(7): 1978-1992. doi: 10.1016/j.apsb.2021.03.035 34386332 PMC8343192

[34] Chen Q, Hu Q, Dukhovlinova E, et al. Photothermal therapy promotes tumor infiltration and antitumor activity of CAR T cells. Adv Mater, 2019, 31(23): e1900192. doi: 10.1002/adma.201900192 30916367 PMC7262962

[35] Zhu L, Liu J, Zhou G, et al. Remodeling of tumor microenvironment by tumor-targeting nanozymes enhances immune activation of CAR T cells for combination therapy. Small, 2021, 17(43): e2102624. doi: 10.1002/smll.202102624 34378338

[36] Ma W, Zhu D, Li J, et al. Coating biomimetic nanoparticles with chimeric antigen receptor T cell-membrane provides high specificity for hepatocellular carcinoma photothermal therapy treatment. Theranostics, 2020, 10(3): 1281-1295. doi: 10.7150/thno.40291 31938065 PMC6956810

[37] Miller IC, Zamat A, Sun LK, et al. Enhanced intratumoural activity of CAR T cells engineered to produce immunomodulators under photothermal control. Nat Biomed Eng, 2021, 5(11): 1348-1359. doi: 10.1038/s41551-021-00781-2 34385695 PMC8791016

[38] Nguyen NT, Huang K, Zeng H, et al. Nano-optogenetic engineering of CAR T cells for precision immunotherapy with enhanced safety. Nat Nanotechnol, 2021, 16(12): 1424-1434. doi: 10.1038/s41565-021-00982-5 34697491 PMC8678207

